# Reconciling the object and spatial processing views of the perirhinal cortex through task‐relevant unitization

**DOI:** 10.1002/hipo.23304

**Published:** 2021-02-01

**Authors:** Julien Fiorilli, Jeroen J. Bos, Xenia Grande, Judith Lim, Emrah Düzel, Cyriel M. A. Pennartz

**Affiliations:** ^1^ Cognitive and Systems Neuroscience Group, SILS Center for Neuroscience University of Amsterdam Amsterdam The Netherlands; ^2^ Research Priority Area Brain and Cognition University of Amsterdam Amsterdam The Netherlands; ^3^ Donders Institute for Brain, Cognition and Behavior Radboud University and Radboud University Medical Centre Nijmegen The Netherlands; ^4^ Institute of Cognitive Neurology and Dementia Research Otto‐von‐Guericke University Magdeburg Magdeburg Germany; ^5^ German Center for Neurodegenerative Diseases Magdeburg Germany; ^6^ Institute of Cognitive Neuroscience University College London London UK

**Keywords:** contextual processing, hippocampus, multisensory integration, perirhinal cortex, spatial coding

## Abstract

The perirhinal cortex is situated on the border between sensory association cortex and the hippocampal formation. It serves an important function as a transition area between the sensory neocortex and the medial temporal lobe. While the perirhinal cortex has traditionally been associated with object coding and the “what” pathway of the temporal lobe, current evidence suggests a broader function of the perirhinal cortex in solving feature ambiguity and processing complex stimuli. Besides fulfilling functions in object coding, recent neurophysiological findings in freely moving rodents indicate that the perirhinal cortex also contributes to spatial and contextual processing beyond individual sensory modalities. Here, we address how these two opposing views on perirhinal cortex—the object‐centered and spatial‐contextual processing hypotheses—may be reconciled. The perirhinal cortex is consistently recruited when different features can be merged perceptually or conceptually into a single entity. Features that are unitized in these entities include object information from multiple sensory domains, reward associations, semantic features and spatial/contextual associations. We propose that the same perirhinal network circuits can be flexibly deployed for multiple cognitive functions, such that the perirhinal cortex performs similar unitization operations on different types of information, depending on behavioral demands and ranging from the object‐related domain to spatial, contextual and semantic information.

## 1 INTRODUCTION

**FIGURE 1:**
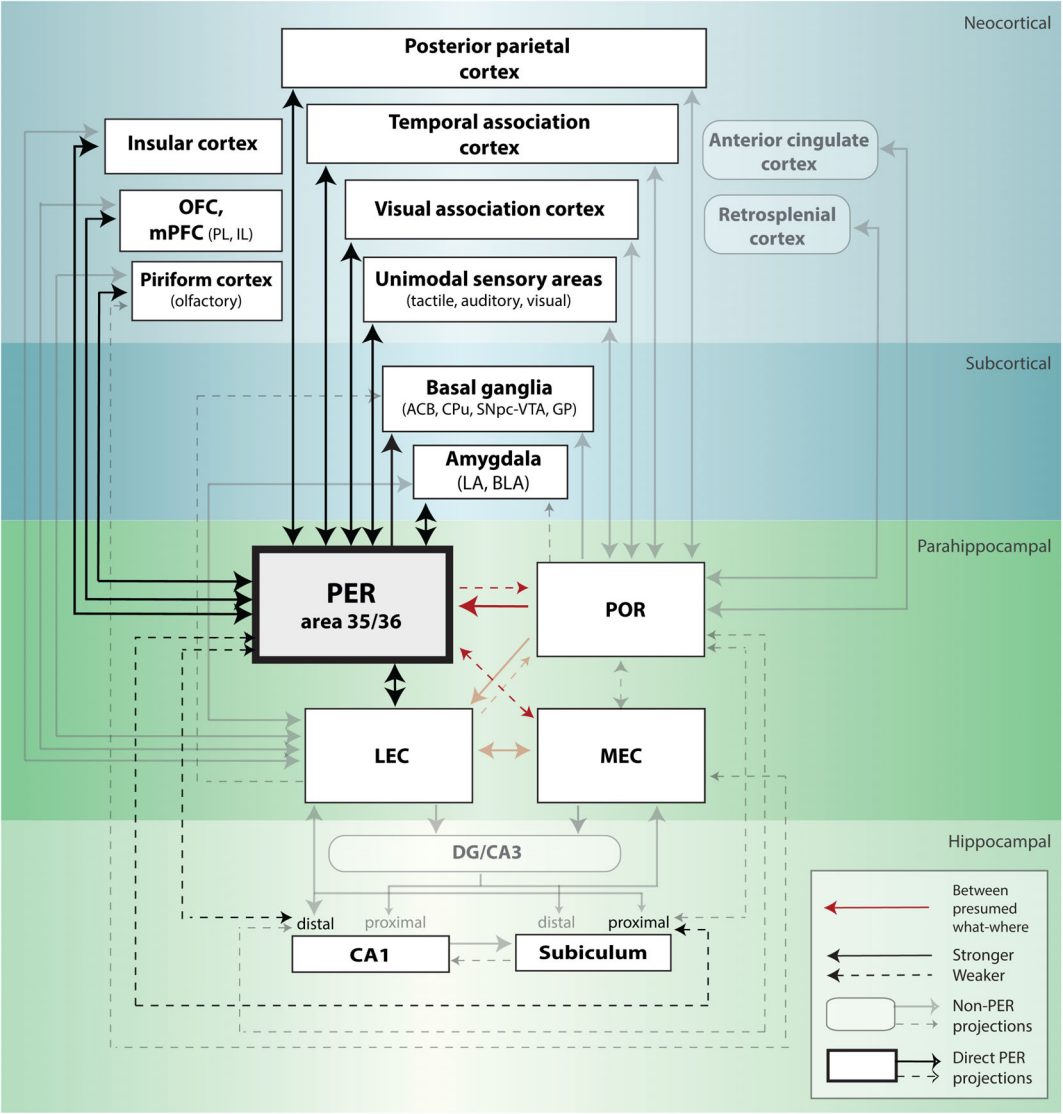
Schematic overview of main anatomical connections to and from the perirhinal cortex in the rat brain. Reappraisal of parahippocampal connectivity has led to an updated anatomical wiring scheme incompatible with a strict “what” versus “where” dichotomy. Notably, LEC is targeted by both POR and PER. PER receives direct and dense input from both POR and LEC, and also has direct reciprocal connections with MEC, CA1, and subiculum. The PER can be differentiated from other parahippocampal regions by appreciating its wide variety of direct connections with areas central in sensory, spatial, motivational and emotional processing (i.e., amygdala, OFC, mPFC, and unimodal sensory areas). Connections between areas are indicated schematically as arrows with uniform or dashed lines for relatively strong and weak projections, respectively. Dark arrows (and associated regions) indicate direct projections to and from the PER. Other connections and areas are indicated as semi‐transparent. Note that many weaker connections to or from non‐PER areas are not included, for example, neocortical projections to the LEC and MEC (schematic connectivity based on Burwell, 2000; Agster & Burwell, 2009; Doan, Lagartos‐Donate, Nilssen, Ohara, & Witter, 2019, Nilssen, Doan, Nigro, Ohara, & Witter, 2019; OFC, orbitofrontal cortex; mPFC, medial prefrontal cortex; amygdala (LA, lateral nucleus of the amygdala; BLA, basolateral nucleus of the amygdala); POR, postrhinal cortex; PER, perirhinal cortex; LEC, lateral entorhinal cortex; MEC, medial entorhinal cortex; DG, dentate gyrus; CA1, cornu ammonis 1; CA3, cornu ammonis 3; PL, prelimbic; IL, infralimbic; VTA, ventral tegmental area; GP, globus pallidus; CPu, caudate putamen; SNpc, substantia nigra pars compacta; ACB, accumbens nucleus)

The perirhinal cortex (PER) is situated at the border between the higher sensory cortices and the entorhinal‐hippocampal complex. On the one hand, the PER can be characterized as a polymodal association area, receiving inputs from many uni‐ and polysensory areas (Burwell, 2001; Burwell & Amaral, 1998a; Burwell & Amaral, 1998b; Burwell, Witter, & Amaral, 1995; Furtak, Wei, Agster, & Burwell, 2007; Suzuki & Amaral, 1994). On the other hand, it is an input and output hub of the medial temporal lobe (MTL), having direct and indirect connections with the hippocampus (HPC) (Burwell et al., 1995; Burwell & Amaral, 1998a; Burwell & Amaral, 1998b; Insausti, Herrero, & Witter, 1997; Witter, Naber, et al., 2000). PER has been defined as consisting of cytoarchitecturally defined Brodmann areas 35 and 36. In the rat, Area 35 receives most projections from the entorhinal cortex (EC), piriform cortex, insular cortex and the amygdala. Area 36 receives major projections from temporal association areas, insular cortex, EC and amygdala (Figure 1; Burwell et al., 1995; Furtak et al., 2007; Agster, Tomás Pereira, Saddoris, & Burwell, 2016; Tomás Pereira, Agster, & Burwell, 2016; Burwell, 2000). The PER has return projections to all of these input areas. The PER is traditionally considered part of a cortico‐hippocampal pathway that is associated with object coding (the “what” pathway), routing this information to the HPC via the lateral EC (LEC). A second pathway, related to spatial coding, (the “where” pathway) has been proposed to comprise the postrhinal cortex (POR), projecting to the medial EC (MEC), which in turn connects to the HPC (Burwell, 2000; Furtak et al., 2007; Goodale & Milner, 1992; Knierim, Neunuebel, & Deshmukh, 2014; Otto & Eichenbaum, 1992; Witter, Wouterlood, Naber, & Haeften, 2000). More recently, however, this strict anatomical dissociation has been disputed (Agster & Burwell, 2009; Doan et al., 2019; Nilssen et al., 2019). Additionally, direct reciprocal connections have been described between PER, the distal CA1, and proximal subiculum (Naber, Silva, & Witter, 2001).

The classical distinction between a “what” and a “where” pathway has been supported by evidence for spatial information coded by grid cells in MEC and the absence of spatial correlates in LEC, that is in the absence of objects (Hafting, Fyhn, Molden, Moser, & Moser, 2005, McNaughton, Battaglia, Jensen, Moser, & Moser, 2006; Knierim et al., 2014; but see below for PER). Important evidence for a perirhinal function in object processing comes from PER lesion studies in rats, resulting in impairments in delayed object recognition (Albasser et al., 2015; Bartko, Winters, Cowell, Saksida, & Bussey, 2007; Ennaceur & Aggleton, 1997; Ennaceur, Neave, & Aggleton, 1996; Norman & Eacott, 2005; Otto & Eichenbaum, 1992). Moreover, single unit recordings in the PER of monkeys and rodents demonstrated that PER neurons are sensitive to the prior presentation of objects, typically showing decreased activation with repeated presentations (Brown & Banks, 2015; von Linstow Roloff, Muller, & Brown, 2016; Young, Otto, Fox, & Eichenbaum, 1997; Zhu & Brown, 1995).

In addition to its role in object recognition, the PER has been suggested to be important for solving feature ambiguity (Buckley & Gaffan, 1998; Buffalo et al., 1999; Bussey & Saksida, 2005; Bussey & Saksida, 2007; Bussey, Saksida, & Murray, 2002; Meunier, Bachevalier, Mishkin, & Murray, 1993; Saksida, Bussey, Buckmaster, & Murray, 2006; Saksida, Bussey, Buckmaster, & Murray, 2007) and for the processing of complex stimuli, both within and across different sensory modalities (Bartko et al., 2007; Feinberg, Allen, Ly, & Fortin, 2012; Jacklin, Cloke, Potvin, Garrett, & Winters, 2016; Kent & Brown, 2012; Ramos, 2016). More recently, work in rodents and monkeys also suggested that the PER processes information on task‐related context, either spatial or temporal (Bos et al., 2017; Eradath, Mogami, Wang, & Tanaka, 2015; Keene et al., 2016), in line with studies reporting a much denser interconnectivity between the traditionally segregated information‐specific pathways (Figure 1; Kerr, Agster, Furtak, & Burwell, 2007; van Strien, Cappaert, & Witter, 2009; Agster & Burwell, 2013; Doan et al., 2019, Nilssen et al., 2019). The main goal of this review is to examine whether the seemingly contradictory findings on object versus spatial‐contextual processing can be reconciled under a broader functional scope for the PER in information processing that emphasizes unitization of task‐relevant information.

## 2 PERIRHINAL CORTEX: SENSORY PROCESSING AND OBJECT RECOGNITION

### 2.1 Lesion studies

Animal and human lesion studies have made important contributions to our understanding of the neural basis of behavior. The strength of lesion studies lies in the provision of causal evidence on functional contributions of specific brain regions to cognitive functions. In humans, two principal approaches have been taken. First, focal lesions have been investigated that overlap anatomically with the PER. Here, drawing specific conclusions on PER functionality is difficult as these lesions are mostly unilateral (and therefore cause limited impairment) or cover more areas than only the PER. Second, widespread neural injury is studied that can be acute in nature (e.g., due to an encephalitis) or caused by progressive neurodegeneration as in the context of Alzheimer's, semantic or frontotemporal dementia. The relationship between PER damage and cognitive impairment then has to be established through quantification of PER integrity (i.e., volume or cortical thickness). Accurate identification of anatomical PER borders is, however, challenging (Berron et al., 2017; Ding & van Hoesen, 2010). Animal models suffer similar difficulties. However, induced lesions allow more control of the focus of the lesioned areas, and reversible lesions allow within‐subject comparisons.

For decades, the tradition of region—function mapping dominated research on PER. One central question was whether the region should be considered perceptual or mnemonic. Moreover, within the field of memory research an intense debate arose about the mnemonic processes (familiarity or recollection) that are served by the HPC versus other MTL regions, including the PER (see, e.g., Brown & Aggleton, 2001; Squire, Stark, & Clark, 2004; Buckley & Gaffan, 2006; Eichenbaum, Yonelinas, & Ranganath, 2007; Wais, 2008; Naya, 2016). This subsection summarizes reported deficits in memory and sensory processing caused by lesions to the PER in the rodent, primate, and human brain.

#### 2.1.1 Lesions in rats

In rodents, object recognition is commonly assessed using a spontaneous object recognition paradigm (Ennaceur & Delacour, 1988; Albasser et al., 2010.) Initially, rats are allowed to explore two objects. In a subsequent recognition phase, one of the two objects is exchanged for a novel one. The innate preference of rodents to explore novel objects over familiar ones is used to assess whether rats recognize objects as familiar or not. Impairments in recognizing objects as familiar are characterized by a failure to preferentially explore the new object over the old one (Albasser et al., 2010; Ennaceur & Delacour, 1988). It is important to emphasize that differences in exploration times reflect object familiarity, and that this might occur without recollection of object identity from episodic memory.

In PER‐lesioned rats, familiarity‐based object recognition is often spared when delays between object sampling and object recognition are short (<10–40 min). With increased delays, however, these animals display impaired familiarity‐based recognition (Ennaceur & Aggleton, 1997; Ennaceur et al., 1996; Norman & Eacott, 2005; Otto & Eichenbaum, 1992). Other studies have shown that impairments can also be present at zero (or very short) delays (Albasser et al., 2015; Bartko et al., 2007). The duration of delays which can be bridged without impairments caused by PER lesions is dependent on the complexity of the stimuli (Norman & Eacott, 2004). PER‐lesioned rats were able to bridge very long delays (24 hr) when presented with distinct objects. If the objects were very similar this duration decreased to less than 5 min. Similarly, Bartko et al. (2007) reported zero‐delay deficits for PER‐lesioned rats in an oddity discrimination task, where physical objects were explicitly manipulated to be perceptually similar. These results suggest that the role of PER in object recognition measured by means of spontaneous exploration is heavily dependent on object complexity and perceptual ambiguity and that impairments can occur in the absence of a heavy memory load.

The described impairments following PER lesioning may be due to deficits in signaling novelty, sensory processing, or retrieval processes. To clarify this, McTighe, Cowell, Winters, Bussey, and Saksida (2010) used a simplified version of the spontaneous recognition task by presenting either two novel or two familiar objects during the recognition phase. As expected, PER‐lesioned rats showed impairments in familiarity‐based recognition. Strikingly, this impairment was caused by reduced exploration of novel objects by PER lesioned rats, rather than increased exploration of familiar objects as one would expect from an impairment in recalling previous object encounters. Instead, the animals either incorrectly recognized new objects as familiar, or failed to signal their novelty. This impairment of familiarity‐based recognition was rescued when the rats were placed in a dark environment during the retention period. McTighe et al. (2010) hypothesize that visual input during the retention period leads to interference in memory for the PER‐lesioned animals. PER lesions thus would induce an increased susceptibility to visual input. This hypothesis has been challenged by other studies that found no or only weak indications of interference (Albasser et al., 2015; Olarte‐Sánchez, Amin, Warburton, & Aggleton, 2015). Differences between the results of these studies may have arisen due to differences in the experimental setups. Studies that found no effect of proactive interference baited the objects to ensure that rats remained exploring objects consecutively, whereas the exploration of rats in McTighe et al. (2010) was driven by mere curiosity. Differences in the extent of PER lesions might also play a role in these inconsistencies (Olarte‐Sánchez et al., 2015).

It can be challenging to disentangle whether mnemonic or perceptual deficits lead to impairments in spontaneous object exploration due to the limited experimental control over sensory input and the inability to repeatedly assess recognition of a familiar stimulus. Other studies have therefore quantified the effect of PER lesions on performance in stimulus discriminations by operant conditioning. Eacott, Machin, and Gaffan (2001) trained rats on approaching one out of two visual stimuli. For simple visual stimuli (rectangle versus square), PER‐lesioned rats were unimpaired, even when the stimuli were warped to increase stimulus resemblance and thereby task difficulty. In contrast, impairments only arose when complex visual stimuli with shared features were used (Eacott et al., 2001). This result indicates that PER cortex is only necessary when different visual features need to be integrated, but not for solving feature ambiguity of just one visual feature. Comparable results were found in tactile, auditory and olfactory tasks (Feinberg et al., 2012; Kent & Brown, 2012; Kholodar‐Smith, Allen, & Brown, 2008; Lindquist, Jarrard, & Brown, 2004; Ramos, 2014, 2016). For instance, PER excitotoxic ablation had no effect on the performance of a whisker‐based tactile discrimination task when discrimination could be achieved by individual tactile features (particle diameter or grain density of sandpaper). This holds even with increased task difficulty due to more similar stimuli. PER lesioned rats only showed deficits when the combination of individual tactile features was crucial to solve the task. By contrast, HPC lesions did not result in any impairment in the same task (Ramos, 2014, 2016). Moreover, PER lesions impair fear conditioning to discontinuous or complex (natural) sounds, but not to pure tones (Lindquist et al., 2004; Kholodar‐Smith et al., 2008; Kent & Brown, 2012, and Section 3). Together, these studies demonstrate a function of PER for the processing of complex (i.e., consisting of multiple sensory features) but not simple stimuli, independently of the sensory modality. Nonetheless, opposing evidence also exists. Clark, Reinagel, Broadbent, Flister, and Squire (2011) did not find impairments after PER lesions for any condition in a visual discrimination task that gradually morphed stimuli to become increasingly similar perceptually. Murray and Wise (2012) argued that rats could have solved this task by attending to specific parts of the visual stimuli, leaving unresolved to what extent multiple features had been used to categorize the stimuli.

In addition to PER functions in unisensory processing, the convergent innervation of the PER by all sensory cortical systems suggests a potential contribution to multisensory processing (Burwell, 2000; Furtak et al., 2007; Naber, Witter, & da Silva, 2000; Naber, Witter, & Silva, 1999; Witter, Wouterlood, et al., 2000). Causal evidence for the involvement of PER in multisensory processing comes from a cross‐modal version of the spontaneous object recognition paradigm. In this variant, rats either first sample an object in the dark (using their whiskers) and later resample it in the light with a translucent plate placed before the objects (preventing the use of whiskers) or vice versa. Rats that received PER lesions prior to training had deficits in cross‐modal object familiarity‐based recognition, while lesions of the HPC had no effect (Albasser et al., 2010; Reid, Jacklin, & Winters, 2012). In addition to the deficits in cross‐modal familiarity‐based recognition, PER‐lesioned rats showed deficits in visual familiarity‐based recognition (when both initial sampling and later resampling relied on vision). However, these rats were not impaired in the olfactory‐ and/or tactile‐only versions of the same task (Winters & Reid, 2010). Strikingly, multisensory preexposure caused later familiarity‐based recognition to become completely PER‐dependent, even under conditions that were initially PER‐independent. Reversible PER inactivation with lidocaine impaired familiarity‐based recognition in all task variants (cross‐modal, visual, and tactile) compared to control rats with saline injections, even after a single multisensory preexposure. This effect occurred regardless of the timepoint of inactivation during any sampling phase (preexposure or later resampling, Jacklin et al., 2016). Presumably, PER‐mediated neural mechanisms formed a multisensory object representation after the exploration via multiple sensory modalities. These results indicate a functional role of PER in multisensory processing in addition to its role in complex unisensory processing.

#### 2.1.2 Lesions in primates

While the abovementioned studies were conducted in rats, the phylogenetically preserved role of PER in resolving feature ambiguity has been extensively documented for monkeys (Buckley & Gaffan, 1998; Buffalo et al., 1999; Bussey et al., 2002; Bussey & Saksida, 2005; Bussey & Saksida, 2007; Meunier et al., 1993; Saksida et al., 2006; Saksida et al., 2007). Bussey et al. (2002) tested control and PER‐lesioned monkeys in a visual paired associate task. In this task, monkeys were required to discriminate between pairs of pictures and learned to touch reward‐associated pairs while ignoring other pairs. The monkeys were tested in three different conditions. First, in the low‐ambiguity condition of the task, all individual pictures constituting the rewarded pairs were different from the pictures in the unrewarded pairs. Merely remembering the outcome related to individual images is thus sufficient to solve this condition. Second, in high‐ambiguity trials, all individual pictures were both part of one unrewarded and one rewarded pair. Here, a correct choice can only be made based on the combined information of both paired images. Third, in trials with intermediate levels of ambiguity only one picture from each pair was ambiguous. Lesions of the PER severely impaired monkeys in the high‐ambiguity condition and mildly impaired them in the intermediate‐ambiguity condition. They were not impaired in the low‐ambiguity condition. Altogether, rodent and monkey lesion studies indicate that the PER is necessary for reward‐related recognition of object identity, complex stimulus processing and for solving feature ambiguity.

Likewise, the role of the PER has been investigated in human lesion studies. Impairment in memory tasks for humans has been particularly noted when familiarity judgments had to be made about object stimuli or item information (Bowles et al., 2007; Brown & Aggleton, 2001; Buffalo, Reber, & Squire, 1998; Stark & Squire, 2000). Several lesion studies found evidence for specific impairment of object recognition in contrast to recollection, when PER damage was apparent but the HPC was spared (Bowles et al., 2007, 2010; Martin, Bowles, Mirsattari, & Köhler, 2011). Also in terms of perceptual discrimination, object processing specifically seems to be impaired, notably when the complexity and feature ambiguity of the stimuli is high and when integration of multisensory information takes place (Barense, 2005; Barense, Gaffan, & Graham, 2007; Lee, Bussey, et al., 2005; Lee, Buckley, et al., 2005; Lee et al., 2006; Mundy, Downing, Dwyer, Honey, & Graham, 2013; Newsome, Duarte, & Barense, 2012; Taylor, Moss, Stamatakis, & Tyler, 2006). A recent study with rare cases of focal PER lesions confirmed impairment in visual discrimination when feature ambiguity was high. However, memory judgment was unimpaired (Inhoff et al., 2019). Overall, it is still subject to debate whether there is a specific mnemonic in addition to the perceptual function of PER and direct involvement of the human PER in familiarity‐based recognition, but not in recollection (Brown & Aggleton, 2001; Brown & Banks, 2015; Graham, Barense, & Lee, 2010; Squire, Wixted, & Clark, 2007).

The overall picture arising from the lesion literature supports a role of PER in object‐related recognition (mainly familiarity) and perception, particularly multisensory and complex feature integration of objects. Regarding the perceptual and mnemonic functions of the PER, we note that these may be fundamentally entangled because perception is partly driven from memory, at least in many task settings (Graham et al., 2010; Pennatz, 2015; Peterson & Enns, 2015).

### 2.2 Neural correlates

Permanent lesions may cause other brain regions to take over functions of a damaged region. Moreover, behavioral effects alone do not illuminate the neural mechanisms underlying a structure's functions. Therefore, additional evidence for the involvement of the PER in object recognition and sensory processing comes from animal electrophysiology and human neuroimaging studies.

#### 2.2.1 Neural correlates in rats

Single PER units recorded from rats display elevated firing rates in the vicinity of multiple physical objects—defined as discrete and movable objects in a maze or open field environment (Burke, Maurer, et al., 2012; Deshmukh, Johnson, & Knierim, 2012). Neurons in the rat PER can also be sensitive to the prior presentation of objects. These neurons typically decrease their firing rate for repeated exposures to the same stimulus (“repetition suppression,” Zhu & Brown, 1995; Young et al., 1997; Brown & Banks, 2015; von Linstow Roloff et al., 2016, Ahn, Lee, & Lee, 2019). Support comes from immediate early gene studies that show increased c‐fos expression in PER upon exposure to new objects (Zhu, Brown, McCabe, & Aggleton, 1995). This increase was specific to object novelty, as a new environment increased immediate early gene expression only in HPC and not PER. Interestingly, rearranging familiar objects led to c‐fos activations in PER but not in HPC (Aggleton & Brown, 2006; Zhu et al., 1995; Zhu, McCabe, Aggleton, & Brown, 1997). However, repetition suppression is not commonly observed in object exploration in an open field (Burke, Hartzell, Lister, Hoang, & Barnes, 2012; von Linstow Roloff et al., 2016). It has been argued that novelty signals could be fleeting and average out during the relatively long bouts of exploration seen in object recognition tasks, compared to the short and more controlled presentations on stimulus display screens. Another factor influencing repetition suppression effects in PER could lie in reward contingencies (von Linstow Roloff et al., 2016).

To gain insights in the mnemonic and perceptual roles of the PER, Ahn and Lee (2017) recorded PER neurons while rats categorized visual stimuli on a touchscreen as being an egg or a toy figure. Rats were rewarded after correctly categorizing a presented stimulus by touching a disk on the category‐associated left or right side when the stimulus disappeared. Morphing the presented stimuli to achieve various degrees of similarity enabled the authors to quantify whether activity of PER neurons correlated with continuous changes in sensory features of the stimulus, or rather with the animal's perceptual categorization (egg or toy template). In line with a dual role of PER in memory and perception, nearly equal proportions of single units represented the perceptual stimulus feature (degree of warping) and stimulus category (egg or toy).

#### 2.2.2 Neural correlates in primates

Experiments in macaques have addressed how associations between different visual stimuli are encoded in the PER (Fujimichi et al., 2010; Naya, Yoshida, & Miyashita, 2003; Naya, Yoshida, Takeda, Fujimichi, & Miyashita, 2003; Sakai & Miyashita, 1991). In these studies, monkeys were trained to memorize pairs of images. A trial started with the presentation of a cue picture, followed by a delay of a few seconds in which no stimulus was presented. After the delay, two different pictures were presented on the same screen. The monkeys were trained to touch the learned paired associate picture and ignore the distractor picture. Some neurons responded selectively to only one picture of the pair, and showed ramping activity during the delay when the preferred picture was about to be shown as the paired associate. This increase in activity suggested anticipation and recall of the paired image (Sakai & Miyashita, 1991). Other PER neurons responded equally to individual pictures that were paired to each other. These neurons did not distinguish between the two stimuli, but instead only coded the paired stimulus combination (Fujimichi et al., 2010). Individual neurons in the PER thus represented paired stimuli as single integrated items. Coding of stimulus associations in PER can emerge a few days after a stimulus pairing has been learned, suggesting a role for PER in long‐term memory formation (Erickson & Desimone, 1999).

Regarding the human PER, functional neuroimaging studies provide evidence that generally supports and extends the findings from animal and human lesion studies. Supporting evidence for a special bias of the human PER cortex to process item (object and often also face) information is reported throughout the literature (for reviews, see, e.g., Graham et al., 2010; Ranganath & Ritchey, 2012). Related to this type of information, PER activity was functionally modulated when multiple types of information needed to be associated with an item, when new items were encountered, when items had to be (mostly visually) discriminated or when item‐related information had to be retrieved (Awipi & Davachi, 2008; Barense et al., 2007; Barense, Henson, & Graham, 2011; Barense, Henson, Lee, & Graham, 2009; Bowles et al., 2010; Devlin & Price, 2007; Diana, Yonelinas, & Ranganath, 2010; Holdstock, Mayes, Gong, Roberts, & Kapur, 2005; Lee et al., 2006; Lee, Scahill, & Graham, 2008; Martin et al., 2011; Martin, Cowell, Gribble, Wright, & Köhler, 2016; Montaldi, Spencer, Roberts, & Mayes, 2006; Mundy et al., 2013; O'Neil, Barkley, & Köhler, 2013; Staresina & Davachi, 2008; Staresina, Duncan, & Davachi, 2011). O'Neil et al. (2012) demonstrated differential functional connectivity profiles of the PER, depending on whether a task required more perceptual or mnemonic judgments on stimulus material. In the memory task the PER was functionally connected to, a.o. ventrolateral prefrontal, anterior cingulate and posterior cingulate cortices, whereas in the perceptual task functional connectivity was stronger to, a.o. fusiform regions and dorsolateral prefrontal cortex (O'Neil et al., 2012). Indeed, recent accounts of human PER function attempt to move away from a process‐specific dissociation of function and rather attribute a specific representational role for the PER in object‐related information that may serve memory or perception, depending on task requirements (Bastin et al., 2019; Bussey & Saksida, 2005, 2007; Graham et al., 2010; Murray, Bussey, & Saksida, 2007).

Overall, the PER appears to be particularly engaged by complex stimulus material that entails multiple dimensions. Not only the association of attributes (e.g., an adjective associated with an object) and multisensory information (e.g., auditory, visual or tactile features of an object), but also information on the relationship to other objects and object familiarity over the subject's lifetime can increase functional PER activity in humans (Bowles, Duke, Rosenbaum, McRae, & Köhler, 2016; Duke, Martin, Bowles, McRae, & Köhler, 2017; Holdstock, Hocking, Notley, Devlin, & Price, 2009; Staresina & Davachi, 2008; Taylor et al., 2006; Taylor, Stamatakis, & Tyler, 2009; Zeithamova, Manthuruthil, & Preston, 2016). Again, this reflects the richness and multidimensionality of the object information assembled in the PER, which can be utilized for both perceptual and mnemonic functions.

## 3 PERIRHINAL CORTEX: PROCESSING SPATIAL CONTEXT AND TASK CONTINGENCIES

### 3.1 Lesion studies

Over the last decade, lesion studies in rats have provided convincing evidence for the involvement of PER in contextual memory. For instance, the PER was shown to be important for the memorization of contextual fear because bilateral PER lesions reduce freezing behavior in prelesion conditioned spatial contexts, but not in other types of fear conditioning (Bucci, Phillips, & Burwell, 2005; Bucci, Saddoris, & Burwell, 2002; Kent & Brown, 2012). Moreover, Jo and Lee (2010) demonstrated that excitotoxic PER lesions severely impair rats in the acquisition of an object discrimination task that required the correct identification of an object's location (in a multiarm maze). The acquisition of object‐discrimination in itself (i.e., independent of location) was completely spared (see also Bussey, Wise, & Murray, 2001). Here, the acquisition of object discriminations might have been guided by differences in simple features, thereby alleviating the loss of PER.

While at least some consensus exists for a contribution of PER to contextual memory in rats, rodent studies often report only mild or no effects of PER lesions in allocentric spatial reference tasks (Liu & Bilkey, 2001; Ramos, 2013; Wiig & Bilkey, 1994). Ramos and Vaquero (2005) reported no effect of PER lesions on the acquisition and short‐term retention (24 hr) in an allocentric navigation task. However, PER lesioned rats were impaired in retention and relearning of the task after long delays (74 days). This points toward a potential role of PER in long‐term allocentric spatial memory. Abe, Ishida, Nonaka, and Iwasaki (2009) also reported that PER lesions impaired previously learned place discriminations in addition to object discrimination. Postlesion acquisition of new place discriminations was intact, indicating that this functionality is not strictly dependent on the PER, but might only rely on it under certain conditions. Additionally, rats might adapt their navigation strategies after PER lesions. Indeed, PER lesions may bias spatial processing toward egocentric navigation strategies. Ramos (2017) investigated how PER lesions affect spatial strategies in rats navigating to a baited goal arm in a plus maze. In two versions of this task, the goal arm could be initially found by using both allocentric and egocentric navigation strategies. In the first version of the task, the animals could use an alternative egocentric strategy by making body turns at maze junctions instead of allocentric orientation. In the second experiment, rats could use intramaze stimuli such as sandpaper covered floors for egocentric navigation, as well as a large constellation of distal landmarks as allocentric reference points. After task acquisition, navigation strategies were tested by rotating the maze or intramaze cues so that allocentric navigation was required to find the reward. Healthy control rats mainly used allocentric strategies to navigate to the goal in both versions of the task. By contrast, PER lesioned animals predominantly used non‐allocentric strategies. This aligns with a role for PER in allocentric spatial processing and, by the same token, in the integration of different, spatially distributed stimuli into a representation of spatial configuration. Even though PER may facilitate allocentric strategies, it is not unconditionally required for allocentric spatial memory in rats, because other studies report no effect of PER lesioning in allocentric tasks (Liu & Bilkey, 2001; Ramos, 2013; Wiig & Bilkey, 1994). Lesion studies thus indicate that PER is recruited for some specific forms of spatial navigation, but we are not aware of a study that demonstrates a general necessity of PER for spatial navigation.

To our knowledge, spatial processing under focal PER lesions in humans and non‐human primates has not been systematically studied to date. However, perseverance of scene discrimination is reported when the HPC, but not the PER, is affected by dementia‐related pathology in humans (see Graham et al., 2010 for an overview).

### 3.2 Neural correlates

#### 3.2.1 Neural correlates of spatial context in rats

Important empirical evidence for a more complex interaction between the two streams (“what” vs. “where”) is the presence of spatial firing fields in LEC for locations in which rats previously encountered an object (Deshmukh & Knierim, 2011). These firing fields have been shown to be stable for multiple days after an object is removed and thus code for the memorized object location rather than the object itself (Tsao, Moser, & Moser, 2013). This is reminiscent of a mixture of spatial and object coding within the LEC. Single units recorded from PER of rats in an open field or circular arena also show elevated firing rates around one or multiple objects. When an object is added or moved to another location, PER units change their activity patterns to incorporate the changed object, and do not display firing fields on the previous location of an object (Burke, Maurer, et al., 2012; Deshmukh et al., 2012). Thus, in settings without elaborate task contingencies and in relatively simple environments, PER cells display firing fields locked to objects in the environment but not to (allocentric) spatial locations.

In contrast to studies that did not report spatial coding in PER, Bos et al. (2017) found that 72% of PER units showed activations and deactivations locked to spatial segments of a figure‐8 maze. PER units were recorded during a visual discrimination task in which rats were trained to collect a reward on the left or right side‐arm of the maze. Visual stimuli were displayed on two screens in front of the middle arm. During each trial the conditioned stimulus was presented on one screen (e.g., the left screen), while the distractor stimulus was presented on the other (i.e., the right). Rats were rewarded for choosing the side‐arm that was on the same side as the rewarded stimulus and ignoring the other side arm marked by a distractor stimulus. Proximal tactile cues on the side walls of the maze consisted of rough or smooth sandpaper which were pseudo‐randomly exchanged and thus independent of the visual stimuli or side arm. PER neurons displayed sustained activations and deactivations with sharp transitions when the rat navigated from one maze segment to the next. However, they were not affected by choices being correct or incorrect, the spatial location or identity of the visual stimuli, or by the sandpaper structure of the maze walls. These segment‐specific responses also occurred when rats ran in a direction opposing the conventional running direction, supporting the notion that these fields were not a pure reflection of acute sensory input. The boundaries of PER firing fields were better locked to task‐relevant spatial segments (branch points) than simultaneously recorded CA1 place field activity. These responses indicate a role for PER in spatially bounded segmentation of the environment, potentially based on task contingencies.

Similarly, Keene et al. (2016) reported that a significant proportion of PER cells differentiates between spatial contexts rather than mere objects in a context‐guided olfactory association task. Here, rats were trained to dig for reward in one of two cups with scented sand. The cups of sand were positioned in one of two square arenas (contexts) which were linked by an alleyway and with different visual and tactile cues on the walls and floor. The context determined which of the two scented cups was baited with reward. While 16.5% of PER cells differentiated between objects, 29.9% of PER cells differentiated between the two spatial contexts independently of the baited scented objects. Twenty‐eight percent of PER neurons were affected by the combination of object and context. Keene et al. (2016) thus demonstrated that neurons in the PER responded to multiple task components, whether they were object related or contextual.

So far, spatial context modulations by PER have only been reported during tasks with clearly defined task contingencies where stimuli, actions and reward were spatially separated (Bos et al., 2017; Keene et al., 2016). When a task context is absent or simple (e.g., foraging in an open field or spontaneous behavior), activity of PER neurons appears to be mainly related to objects in the environment (Burke, Maurer, et al., 2012, Deshmukh et al., 2012). When tasks become more complex PER neurons get involved in segmenting the environment in task‐relevant chunks (Bos et al., 2017). Another possibility to explain sensitivity to spatial context is that the degree of physical segmentation of the context itself affects the degree of spatial correlates in PER. By making use of physical doors, the environments in experiments from Bos et al. (2017) and Keene et al. (2016) were naturally segmented into different spatial chunks. In contrast, in the studies by Burke, Maurer, et al. (2012) and Deshmukh et al. (2012), complex objects were present in environments which were otherwise homogeneous. Here, the most salient proximal features to encode were the objects. Neural segmentation of the environment may not have occurred due to the circular or open field nature of the maze and/or the lack of spatial task constraints. Thus, from these studies it appears plausible that the degree of physical segmentation of the environment and task demands both play a role in the observed spatial‐contextual responses of PER neurons.

#### 3.2.2 Neural correlates of task contingencies

The relevance of specific task contingencies for PER engagement is supported by multiple reports of PER activity modulations by a wide variety of task rules and reward schedules (Ahn & Lee, 2015; Bos et al., 2017; Kreher et al., 2019; Liu & Richmond, 2000; von Linstow Roloff et al., 2016; Young et al., 1997). Eradath et al. (2015) demonstrated that PER cells in the macaque can represent cue‐outcome associations and the temporal context in which the association occurs. Macaques were trained to associate 12 out of 24 visual cues with a reward while the other 12 cues predicted a sound (but no reward). A single trial consisted of a sequence of two visual cues and outcome. The first part of the trial occurred according to the learned cue‐outcome contingency, whereas in the second part the two types of outcome were randomly provided. PER mainly represented the outcome type (water or sound) contingent on the cue. This representation was dependent on the presentation of visual stimuli, as reward expectancy was not represented by these PER units when rewards were given at predictable time points independently of a visual stimulus. Moreover, PER activity only represented outcome contingency in the trial part where the cue was predictive of reward and not during the randomly rewarded trial epoch, even though the cues during that epoch were previously associated with reward as well. The differentiation started from the onset of the visual cue and remained sustained after reward delivery until the next trial started. This is reminiscent of sustained postreward activations observed in the mPFC, which have been associated with cue‐outcome and action‐outcome learning (Histed, Pasupathy, & Miller, 2009; Mulder, Nordquist, Örgüt, & Pennartz, 2003). When the order of the trial sequence was reversed, the monkeys started to adapt their behavioral outcome expectation within 3 days (i.e., by displaying anticipatory sucking). However, representations of stimulus‐outcome contingencies by PER cells only adapted after 10 days. These results suggest that PER cells represent long‐term memorized cue‐outcome contingencies and the temporal context in which they occurred, rather than purely an expectation of the outcome or expectation of the visual stimulus. Likewise, the behavior of PER lesioned rats in a task that required weighing of delays against reward size suggests a similar role of PER in processing choice‐outcome contingencies in rodents (Kreher et al., 2019).

#### 3.2.3 Neural correlates of landmark and scene processing in humans

In humans, the PER (together with other MTL structures) appears to represent prospective goals in a spatial navigation task (Brown et al., 2016). This may also be interpreted as a type of item‐related representation. Accordingly, functional evidence from a virtual reality study suggests a role of the human PER in wayfinding based on landmarks (Hartley, Maguire, Spiers, & Burgess, 2003). Note, a recent study that shows particularly the posterior part of PER and the human parahippocampal cortex to be associated with landmark‐related object processing (Martin, Sullivan, Wright, & Köhler, 2018). A functional role of the PER specifically in navigation or spatial context has, to our knowledge, not been investigated more extensively in humans. The clear association of the PER with rich and complex object representations does not exclude a PER involvement in any scene processing. Here, scene processing does not relate to navigation directly but instead to the processing of pictures that contain scene information (cf. Berron et al., 2018; Ross, Sadil, Wilson, & Cowell, 2018). While such stimuli clearly engage spatial networks in humans, one may also argue that these scene pictures may be processed as one entity or object. In fact, integration of objects with their context is a key step in interpreting complex visual scenes. Additionally, any evidence about PER function and bias for a specific type of information is influenced heavily by task design. For instance, some fMRI studies report PER activity in relation to scenes by comparing functional activity in PER with a baseline condition (Berron et al., 2018; Ross, Sadil, Wilson, & Cowell, 2018). However, when they compared object with scene conditions, more PER activity in the object condition was shown. Based on human imaging studies we may conclude that the PER is biased toward object processing, and that its involvement in landmark‐based wayfinding and scene processing cannot be excluded.

The abovementioned results, mainly from animal studies, indicate that activity patterns from PER neurons reflect more than object recognition signals or object percepts alone. Instead, the PER supports a much broader variety of representations that seem to be dependent on the cognitive task at hand. This coheres with the attribution of a role in semantic meaning to the human PER, as we outline in the following section.

## 4 A SYNTHESIS OF THE OBJECT VERSUS SPATIAL PROCESSING VIEWS OF THE PERIRHINAL CORTEX

Even though two main functional pathways have been previously delineated—one consisting of the PER‐LEC and the other of the POR‐MEC network, associated with object and spatial coding, respectively—it has become clear that this dissociation falls short of fully capturing the functional role of individual structures such as the PER. The foregoing overview points to the importance of the PER in processing complex and ambiguous stimuli rather than processing any object per se; processing sensory information from simple objects can occur without causal dependence on the PER. Additionally, neurons in the PER can represent a wide variety of learned constructs such as spatial context, temporal context and task contingencies (Ahn & Lee, 2015; Bos et al., 2017; Eradath et al., 2015; Keene et al., 2016; Liu & Richmond, 2000; von Linstow Roloff et al., 2016; Young et al., 1997). The modulation of PER by task contingencies can be understood by taking into consideration the dense anatomical connectivity between PER and motivational structures such as the ventral tegmental area, ventral striatum, amygdala, and orbitofrontal cortex (Figure 1; Agster et al., 2016; McIntyre, Kelly, & Staines, 1996; Witter & Groenewegen, 1986; Pikkarainen & Pitkänen, 2001). The reciprocal functional and anatomical connections between PER, medial prefrontal and orbitofrontal cortex likely convey information on task rules and predicted value of cues, action, and context (Agster & Burwell, 2009; Burwell & Amaral, 1998a; Deacon, Eichenbaum, Rosenberg, & Eckmann, 1983; Delatour & Witter, 2002; McIntyre et al., 1996; Rusu & Pennartz, 2020; Sesack, Deutch, Roth, & Bunney, 1989; van Wingerden, Vinck, Lankelma, & Pennartz, 2010a, 2010b).

Over recent years, it has become clear that there is ample crosstalk between the traditional “what” and “where” streams. PER is reciprocally connected to the LEC, but also receives projections from the “where” pathway predominantly through the POR (Burwell, 2000; Burwell & Amaral, 1998a; Burwell & Amaral, 1998b). POR additionally projects to the MEC and to the dorsolateral LEC (Burwell, 2000; Burwell & Amaral, 1998a; Burwell & Amaral, 1998b; Doan et al., 2019; Kerr et al., 2007). The POR receives modest projections from the presumed “what” pathway through the PER and LEC (Burwell, 2000; Burwell & Amaral, 1998a; Burwell & Amaral, 1998b; Kerr et al., 2007). Thus, the parahippocampal–hippocampal organization includes numerous cross‐connections between the presumed “where” and “what” pathways (Figure 1; cf. Burwell, 2000; Nilssen et al., 2019; van Strien et al., 2009). Additionally, PER has direct and reciprocal connections with CA1 and subiculum (similarly to POR, although mirroring their distal/proximal CA1 target axis; Liu & Bilkey, 1996; Naber et al., 2000; Agster & Burwell, 2013). Furthermore, the crosstalk is evident from spatial firing patterns that have been found in the LEC (Connor & Knierim, 2017; Deshmukh & Knierim, 2011; Knierim et al., 2014; Neunuebel, Yoganarasimha, Rao, & Knierim, 2013; Yoganarasimha, Rao, & Knierim, 2011) and the PER responses related to spatial context (see above; Keene et al., 2016; Bos et al., 2017). Finally, ablating PER reduces HPC place field stability across delays and reduces modulation of place cells by movement (Muir & Bilkey, 2001, 2003).

An alternative hypothesis about the difference between the PER‐LEC and POR‐MEC pathways (instead of object vs. location) is a distinction in coding for proximal versus distal spatial cues, respectively (Knierim et al., 2014; Neunuebel et al., 2013). Activity patterns of LEC neurons preferably rotate with local cues, while activity patterns of MEC neurons follow spatial rotations of distal cues. However, to date, no double cue rotation studies have been performed in combination with recordings from PER. Moreover, as for the “what” versus “where” dissociation, the proximal versus distal dichotomy cannot account for spatial‐contextual coding by the PER (Ahn & Lee, 2015; Bos et al., 2017; Eradath et al., 2015). Others have proposed that PER is mainly engaged in processing fine‐grained information of relevant stimuli (Burke et al., 2018). This proposal aligns with most abovementioned deficits in processing task‐relevant and complex stimuli following PER lesions. It is, however, somewhat difficult to reconcile with the irrelevance of intact PER for very precise (detailed) perceptual discriminations based on individual sensory features (Eacott et al., 2001; Feinberg et al., 2012; Kent & Brown, 2012; Kholodar‐Smith et al., 2008; Lindquist et al., 2004; Ramos, 2014, 2016).

Overall, the functional role of the PER appears to mainly be related to the complexity of task‐relevant information. The rodent and monkey literature in particular emphasize the importance of the PER for the representation of complex stimuli in which diverse spatial‐contextual features have to be integrated, but not of simple stimuli. One way to reconcile the results on object‐ versus spatial‐contextual coding is by building on the proposal that the PER is recruited when different features can be merged perceptually or conceptually into a single entity (Bang & Brown, 2009; Bussey & Saksida, 2007; Ho & Burwell, 2014; Kent & Brown, 2012; Kent, Hvoslef‐Eide, Saksida, & Bussey, 2016; Kholodar‐Smith et al., 2008; Ranganath & Ritchey, 2012; Suzuki & Naya, 2014). Combining different features into a single entity is referred to as unitization (Graf & Schacter, 1989), and was initially proposed to be a main function of the PER in the context of fear conditioning (Kent & Brown, 2012). Adopting this broader function of the PER in unitization captures the traditional object‐oriented nature of PER, but also PER response patterns emerging in specific spatial task contexts.

**FIGURE 2:**
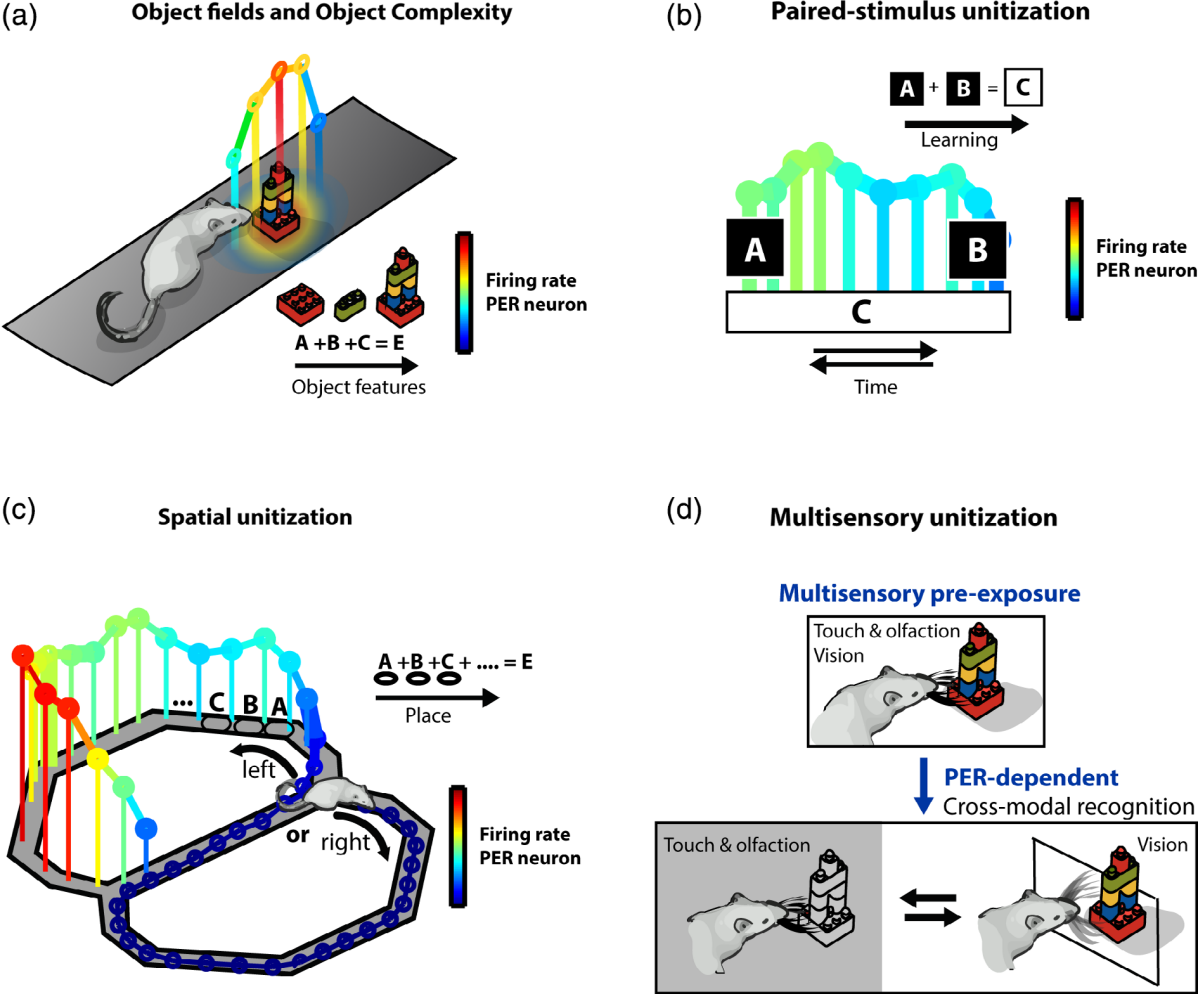
Task‐dependent unitization in the perirhinal cortex. (a) Schematic illustration of reported single unit activity related to object presence in an open field or circular arena (Burke, Hartzell, et al., 2012; Burke, Maurer, et al., 2012; Deshmukh et al., 2012). PER is mainly necessary in spontaneous object recognition tasks for complex objects, but less for simple ones. This suggest a functional role of PER for the unitization of complex object features (Bartko et al., 2007; Norman & Eacott, 2004; Ramos, 2014, 2016). (b) During a visual paired‐associate task, neurons in the macaque PER encode the learned pairings between stimuli in a unitized manner over time (Fujimichi et al., 2010). (c) PER neurons of rats performing a visual discrimination task on a figure‐8 maze display sustained responses associated with different maze segments (Bos et al., 2017). This suggests a spatial unitization relative to finer forms of spatial coding as found in dorsal hippocampal area CA1. (d) A reversible lesion study demonstrated that multimodal preexposure increases the importance of PER in later cross‐modal object recognition. This could potentially result from the formation of a unitized (multisensory) representation of the object (Jacklin et al., 2016)

This functionality definition circumvents an overly strict distinction between spatial and object processing which is difficult to maintain conceptually. For instance, is a house, street or neighborhood within a city considered to be “spatial” or do these entities constitute an “object” figuring in a “what” pathway? In other words, the distinction between spatial descriptors and objects is not conceptually unambiguous and depends on the cognitive task at hand. Within a homogeneous open field or linear environment, there are no discrete spatial “chunks,” standing out a priori. In increasingly complex environments, sensory discriminants can be used to parse the complex space into simple chunks. Spatial processing may then benefit from unitization by integrating small subspaces of an environment into larger units, corresponding to large spatial fields (Bos et al., 2017). This is illustrated by the integration of assemblies of buildings and landmarks into larger chunks—for example, residential blocks, streets or neighborhoods—which can be subsequently used to avoid the curse of dimensionality when choosing an action policy for complex tasks (Pezzulo, van der Meer, Lansink, & Pennartz, 2014). Unitization can also be applied to other types of information processing, such as the processing of discrete events in time which have a shared behavioral relevance (Figure 2).

This reconceptualization of the PER can be extended to the interpretation of results from human studies. Indeed, some fMRI and lesion studies in humans as well as the computational model by Bussey and Saksida et al. (2007) point to the idea that feature ambiguity can be solved efficiently by unitization. Furthermore, functional activity changes in the PER indicate unitized representations in that region (Bussey & Saksida, 2007; Cowell, Bussey, & Saksida, 2006; D'Angelo, Noly‐Gandon, Kacollja, Barense, & Ryan, 2017; Delhaye, Tibon, Gronau, Levy, & Bastin, 2018; Diana et al., 2010; Fujimichi et al., 2010; Haskins, Yonelinas, Quamme, & Ranganath, 2008; O'Neil et al., 2013; Rubin, Chesney, Cohen, & Gonsalves, 2013; Taylor et al., 2009). Still, directly supporting evidence is mixed. The PER was more functionally active when unitization of words had to be carried out, for example, in a condition where compound words had to be explicitly built versus a condition in which single words had to be entered into an associative sentence (Haskins et al., 2008). PER damage affected performance in tasks that benefit from unitization of stimulus components (Delhaye, Bahri, Salmon, & Bastin, 2019). Furthermore, adopting a unitization strategy (i.e., encoding information by creating binding relations between elements) facilitated performance of amnesic patients with hippocampal damage in an associative memory paradigm (D'Angelo, Kacollja, Rabin, Rosenbaum, & Ryan, 2015). However, in another study, the unitization of visual stimuli was not directly associated with a BOLD increase in PER activity (Staresina & Davachi, 2010). In this study, participants had to encode pictures of objects. In some conditions, these objects were displayed as being cut into two or four pieces and arranged such that they did not appear as a coherent object because each piece was attached and tilted toward the sides of the screen. In this condition, the objects needed to be visually integrated. While PER activity parametrically increased when more item‐related information was encoded, the demand of the unitization condition (e.g., whether the item was cut in multiple pieces) did not modulate PER activity. The authors speculate as to whether the PER may be involved in the unitization process itself or whether the PER exploits other functions using unitized representations.

We note that there may be differences between mere perceptual, imagery‐based and conceptual unitization (Rubin et al., 2013; Staresina & Davachi, 2010). In Staresina and Davachi's experiment, participants needed to form perceptual units by moving the pieces of objects mentally together (i.e., using visual imagery); however, no new concept was created (see also Delhaye et al., 2019). In contrast to such unitization by imagery‐from‐perception, Haskins et al. (2008) required participants to unitize the meaning of two words and thereby form a new conceptual entity (e.g., “book” and “worm” becomes “bookworm”). The latter thus refers to a higher‐level cognitive task that unitizes information by attributing (new) meaning. Presumably, the human PER is specifically involved in the latter. Indeed, human fMRI studies on the PER stress its particular involvement when semantic meaning is relevant (for a related conceptual model see (Miyashita, 2019). Specifically, the medial human PER (segmented following Taylor & Probst, 2008; but note Ding & van Hoesen, 2010; Berron et al., 2017) may help to dissociate confusable objects, for example, objects that share perceptual features but also meanings (Kivisaari, Tyler, Monsch, & Taylor, 2012). These results align with a stream of human data indicating a PER function in semantic cognition. Conceptual learning, semantic processing and semantic priming have been associated with PER functional activity and PER damage leads to an inability in making fine semantic discriminations (Bowles et al., 2016; Bruffaerts et al., 2013; Clarke & Tyler, 2014, 2015; Dew & Cabeza, 2013; Kivisaari et al., 2012; Ranganath & Ritchey, 2012; Taylor et al., 2006; Tyler et al., 2004; Wang, Ranganath, & Yonelinas, 2014; Wright, Randall, Clarke, & Tyler, 2015). For example, Bruffaerts et al. (2013) presented words that were previously clustered semantically and whose semantic distances were determined. Interestingly, when analyzing multivariate representational similarities of the fMRI voxel patterns evoked by presented words, the PER reflected the semantic distances. That is, words with a more distinct meaning were associated with a multivoxel activity pattern that was likewise more distinct and vice versa. This ability to make semantic discriminations is compromised by PER damage and related to PER functional activity in healthy humans (Tyler et al., 2004). Moreover, prior exposition to a semantically similar word usually improves performance on a memory task that uses conceptual retrieval cues (“conceptual priming”). Conceptual priming is impaired by extended MTL lesions that incorporate the PER and, in healthy participants, conceptual priming is associated with an increase in PER activity (Wang et al., 2014; Wang, Lazzara, Ranganath, Knight, & Yonelinas, 2010). These human data add to PER functions in unitization by suggesting a multidimensional role in integrating conceptual information attributed to encountered objects.

It may appear as a contradiction that semantic discrimination as well as unitization are attributed to PER function. Upon closer examination, however, both functions are in fact well compatible and may be based on the same underlying principle: unitization of information may eventually support fine semantic discrimination (Bastin et al., 2019; Cate & Köhler, 2006; Kent et al., 2016). For instance, semantically similar items also exhibit feature ambiguity (in semantic terms or perceptual terms). Unitization integrates diverse information into one entity that exceeds the amount of integration expressed by its parts (e.g., “white house” contains more information than “white” and “house” separately). This greater specificity may enable better fine discrimination. However, the rules by which certain elements are lumped together still have to be determined and may relate to current task goals. It remains to be examined whether the PER is indeed involved in these higher‐level unitization processes or rather uses unitized representations for further processing.

## 5 DISCUSSION

Although neural correlates of object recognition and familiarity have been documented in rodents, primates and humans, empirical evidence also points toward a contribution of PER in associating and unitizing a wide variety of other types of task‐relevant information. Nonetheless, there still exists uncertainty on the extent and precise conditions of PER contributions to various types of integration. Apparent inconsistencies across the PER literature may be related to the influence of task demands on PER activity. Bos et al. (2017), for instance, also found object responses besides the previously reported spatial responses (sometimes even of the same cell). The degree to which PER codes for object or spatial information may thus greatly vary from one task to the other, in line with a more general role in unitizing meaningful entities. Reported inconsistencies between empirical results from studies investigating the PER might also partly be attributable to differences between targeted PER subregions, or in human fMRI to averaging over different subdivisions. Differences in functional and anatomical connectivity profiles between area 36 and 35 have been described in rodents and monkeys (Burwell, 2001; Burwell et al., 1995; Burwell & Amaral, 1998a; Burwell & Amaral, 1998b; Deacon et al., 1983; Furtak et al., 2007). In fact, Fujimichi et al. (2010) reported an increasing strengthened integration of two paired visual stimuli when going up the cortical hierarchy of the macaque PER, leading from area 36 to area 35. Hints for a functional gradient also exist in humans (Kafkas et al., 2017; Liang, Wagner, & Preston, 2013; Litman, Awipi, & Davachi, 2009; Zhuo et al., 2016). Additionally, differential susceptibility for neurodegeneration (tau pathology) is evident in more lateral PER versus the transition between PER and EC (taking into account the variability in anatomical nomenclature; Braak & Braak, 1991; Kaufman, del Tredici, Thomas, Braak, & Diamond, 2018; Berron et al., 2019; Maass et al., 2019). Accurate segmentation of PER in human fMRI studies, particularly the delineation of the border between area 35 and 36, is challenging (Braak & Braak, 1991; Ding & van Hoesen, 2010; Kivisaari et al., 2012). In human fMRI, this border is often defined by the collateral sulcus that exploits a considerable variety in anatomical expression between individuals (and also hemispheres; Ding & van Hoesen, 2010). More precise studies on human PER function are expected as only recently methodological advances have been made in high‐resolution imaging to segment the structure with a fundamentally higher level of detail, also delineating PER subdivisions (Berron et al., 2017).

Irrespective of the anatomical subdivisions within the PER, this cortical region is thought to act as a transition or gateway area between the neocortex and hippocampal system. However, instead of being a passive gateway, the PER is often portrayed as an “inhibitory wall,” determining which neocortical information is conveyed to the hippocampal system via the LEC (Biella, Uva, & de Curtis, 2001, 2002; de Curtis & Paré, 2004; Martina, Royer, & Paré, 2001; Nilssen et al., 2019; Pelletier, Apergis, & Paré, 2004; Willems, Wadman, & Cappaert, 2016). This gating mechanism has not been widely investigated in relation to its integrative or unitizing functions. Convergent input from different distant regions may be required to reduce local inhibition in the PER to allow response transmission from the PER to the LEC and HPC (de Curtis & Paré, 2004; Unal, John, & Paré, 2012; Nilssen et al., 2019; see also below). This gating pattern may facilitate task‐dependent integrative functions of PER required for both object recognition and spatial‐contextual processing. To select which task‐relevant information is transferred to the hippocampal system, a gating system such as PER needs information on, for instance, which elements from the sensorium belong together and are collectively predictive of outcome (e.g., reward) and which do not. In that sense, task‐relevant mnemonic gating without any form of unitization seems difficult to realize. The interactions between gating and execution of unitizing operations in PER remain to be investigated, as well as how PER gating and firing may depend on hippocampal feedback, which in behaving animals may be expressed in phase locking of PER neurons to the hippocampal theta rhythm (Ahn et al., 2019; Bos et al., 2017).

The gating property of the PER may provide an intrinsic mechanism for the integrative PER functions, but does not directly explain observed differences between anterograde and retrograde memory disruptions after PER lesions. For instance, PER‐lesioned rats were impaired in previously learned object discriminations (retrograde amnesia) but could relearn to discriminate between new objects after the lesions (Jo & Lee, 2010). Surprisingly, however, the same rats were unable to relearn new object‐place associations. Similarly, multisensory object preexposure causes later familiarity‐based recognition to be more dependent on the PER (Jacklin et al., 2016). These lesion effects indicate that stored representations are often lost after PER lesions, but that relearning can take place, possibly based on simpler (non‐ambiguous, e.g., luminosity‐ or contrast‐based) sensory features. Intriguingly, retrograde loss of object‐place associations due to PER lesions could be compensated after some days of retraining, whereas new object‐place associations could not be acquired. It has been proposed that memorized associations are encoded by distributed cortical assemblies across different cortical regions, which are recruited during successful memory retrieval. The ability to relearn lost associations between objects and locations after PER lesioning, but not to gain new ones, hints at assemblies outside the PER being sculpted by PER activity during learning (also see Doron et al., 2020).

The importance of PER for storing unitized representations raises questions about the underlying computational mechanisms. In this respect we can advance two hypotheses which may account for unitization of more elementary representations. First, it has been proposed before that the parahippocampal–hippocampal system may harbor plastic, recurrent networks that may implement autoassociative memories with pattern‐completing capacities (e.g., in area CA3; Hopfield, 1982; Treves & Rolls, 1992; Nakazawa et al., 2002; Grande et al., 2019) as well as pattern‐separating networks (especially associated with the dentate gyrus; Treves & Rolls, 1992, Leutgeb, Leutgeb, Moser, & Moser, 2007; Neunuebel & Knierim, 2012, Berron et al., 2016). Autoassociative memories are conventionally rendered as simple, amodal patterns and can be fully retrieved by offering partial cue information. We propose that their active reinstatement in, for example, HPC leads to information transmission to downstream areas including LEC and PER, where multiple simple representations may be merged into more complex ones, possibly involving acute bottom‐up input from the sensory neocortices. Second, unitization can also be understood as an extended form of predictive processing, based on the tenet that lower sensory cortices reciprocally interact with higher areas to generate predictive representations as a way of modeling the causes of sensory input (Friston, 2005; Pennartz, Dora, Muckli, & Lorteije, 2019; Rao & Ballard, 1999). Whereas primary sensory cortices may be concerned with modeling causes of simple sensory patterns (e.g., a local patch with an oriented grating in the visual field), we hypothesize that higher sensory areas may interact with PER and other parahippocampal structures to integrate low‐level predictions into high‐level representations that combine features within and across modalities (Olcese, Oude Lohuis, & CMA, 2018; Struckmeier et al., 2019). These unitized representations may both influence, and be influenced by, hippocampal patterns characterized by a yet stronger form of invariance, expressed in allocentric and conceptual representations (Buzsáki & Moser, 2013; O'Keefe & Dostrovsky, 1971; Pennatz, 2015; Quiroga, 2012). We stress that these two computational hypotheses are not mutually exclusive and that PER may harbor additional computational functions, such as novelty filtering (Haltsonen, Jalanko, Bry, & Kohonen, 1978). Returning to the “gating” function of the PER, both computational paradigms allow to hypothesize that unitized representations, stored as such in the PER and its closely connected structures, may be evoked and reinstated when sufficient bottom‐up sensory evidence is presented through lower‐level cortical areas to allow completion of the unitized pattern, which then acts as a prerequisite for further transmission into the hippocampal system. Furthermore, both the associative memory‐merging and predictive processing schemes are well compatible with additional Reinforcement Learning, the effects of which may reach the PER via, for example, mesencephalic dopaminergic, prefrontal, and amygdaloid structures, putatively relying on glutamatergic and/or dopaminergic transmission (Pennartz, 1997; Tomás Pereira et al., 2016). These computational hypotheses will require further development by multiarea computational modeling as well as empirical testing.

In conclusion, experiments addressing the functional role of PER across different species and behavioral paradigms strongly suggest a function for the PER in representing different types of information as unitized entities, thereby reconciling the object‐ and space‐processing views on PER. Rodent work indicates that the PER is primarily object‐oriented in the absence of task demands, but also emphasizes that PER can support representations of spatial context, task contingencies and objects associated with both of these. Neurophysiological studies in macaques further support the notion that the PER unitizes sensory information into meaningful perceptual and conceptual entities, as illustrated by tasks for solving visual feature ambiguity, and by neural representations of learned pairings of visual stimuli or stimulus‐outcome items in the PER. Finally, studies in humans underline that here the PER has become specialized in the unitization of items with related semantics. A promising approach to investigate how PER recruitment depends on task demands will be to record PER neuronal activity under different spatial and contextual task demands (e.g., recording the same neurons in different tasks and environments). Other avenues for future research involve reversible and specific inhibition of PER afferents and relating different task‐dependent neural correlates to distinct PER circuits. Finally, recent methodological advances in structure segmentations for high‐resolution fMRI allow researchers to investigate PER subdivisions separately and examine whether functionality fundamentally distinguishes human PER subdivisions.

## CONFLICT OF INTEREST

The authors declare no conflict of interest.

## Data Availability

Data sharing is not applicable to this article as no new data were created or analyzed in this study.
